# On three-term conjugate gradient method for optimization problems with applications on COVID-19 model and robotic motion control

**DOI:** 10.1186/s13662-021-03638-9

**Published:** 2022-01-04

**Authors:** Ibrahim Mohammed Sulaiman, Maulana Malik, Aliyu Muhammed Awwal, Poom Kumam, Mustafa Mamat, Shadi Al-Ahmad

**Affiliations:** 1 Department of Mathematics and Statistics, School of Quantitative Sciences, College of Art and Sciences (CAS), Universiti Utara Malaysia (UUM), 06010 Sintok, Kedah Malaysia; 2 Department of Mathematics, Universitas Indonesia (UI), Depok, 16424 Indonesia; 3 Department of Mathematics, Faculty of Science, Gombe State University, Gombe, Nigeria; 4 Center of Excellence in Theoretical and Computational Science (TaCS-CoE), Faculty of Science, King Mongkut’s University of Technology Thonburi (KMUTT), 126 Pracha Uthit Rd., Bang Mod, Thung Khru, Bangkok, 10140 Thailand; 5 KMUTT-Fixed Point Theory and Applications Research Group, Theoretical and Computational Science Center (TaCS), Science Laboratory Building, Faculty of Science, King Mongkut’s University of Technology Thonburi (KMUTT), 126 Pracha-Uthit Road, Bang Mod, Thrung Khru, Bangkok, 10140 Thailand; 6 Department of Medical Research, China Medical University Hospital, China Medical University, Taichung, 40402 Taiwan; 7 Faculty of informatics and Computing, Universiti Sultan Zainal Abidin (UniSZA), Terengganu, 22200 Malaysia

**Keywords:** 90C30, 90C06, 90C56, Finite difference, Three-term CG algorithms, Optimization models, Motion control, Line search procedure, Coronavirus (COVID-19), Regression analysis

## Abstract

The three-term conjugate gradient (CG) algorithms are among the efficient variants of CG algorithms for solving optimization models. This is due to their simplicity and low memory requirements. On the other hand, the regression model is one of the statistical relationship models whose solution is obtained using one of the least square methods including the CG-like method. In this paper, we present a modification of a three-term conjugate gradient method for unconstrained optimization models and further establish the global convergence under inexact line search. The proposed method was extended to formulate a regression model for the novel coronavirus (COVID-19). The study considers the globally infected cases from January to October 2020 in parameterizing the model. Preliminary results have shown that the proposed method is promising and produces efficient regression model for COVID-19 pandemic. Also, the method was extended to solve a motion control problem involving a two-joint planar robot.

## Introduction

Consider the following optimization model:

1.1 $$ \min f(x), \quad x \in \mathbb{R}^{n}, $$

where $f:\mathbb{R}^{n} \to \mathbb{R}$ is a smooth function whose gradient $\nabla f(x)=g(x)$ is available. Problems of the form (1.1) can be traced to many professional fields of science, astronomy, engineering, economics, and many more (see, for example, [1, 2]). Throughout this paper, we shall abbreviate $g(x_{k})$ and $f(x_{k})$ by $g_{k}$ and $f_{k}$, respectively. Also, $\|\cdot\|$ represents the Euclidean norm of vectors.

The nonlinear CG methods play an important role in solving large-scale optimization models due to the modesty of their memory requirements and nice convergence properties. Generally, the iterates of the CG methods are usually determined through the following recursive computational scheme:

1.2 $$ x_{k+1}=x_{k}+s_{k}, \quad s_{k}=t_{k} d_{k},\quad k \geq 0, $$

where $t_{k}$ is the step-size computed along the search direction $d_{k}$. For the first iteration, $d_{0}$ is always the steepest descent direction, that is, $d_{0}=-g_{0}$ [3]. However, subsequent directions are recursively determined by

1.3 $$ d_{k}=-g_{k}+\beta _{k} d_{k-1},\quad k\geq 1, $$

where the scalar $\beta _{k}$ is known as the CG coefficient whose different form determines a different CG methods.

The following line search procedures have been used in the convergence analysis and implementations of the already existing CG methods [4]. The convergence analysis often requires the line search to satisfy the exact line search, the Wolfe or strong Wolfe (SWP) line search. The exact line search requires the step-size $t_{k}$ to satisfy

1.4 $$ f(x_{k}+t_{k} d_{k} ):=\min _{t \geq 0} f(x_{k}+t d_{k}). $$

The standard line search requires computing $t_{k}$ such that the cost function is minimized along $d_{k}$ satisfying

1.5 $$\begin{aligned} & f(x_{k}+t_{k} d_{k} )\leq f(x_{k} )+\delta t_{k} g_{k}^{T} d_{k}, \end{aligned}$$

1.6 $$\begin{aligned} & g(x_{k}+t_{k} d_{k})^{T} d_{k}\geq \sigma g_{k}^{T} d_{k}. \end{aligned}$$

The SWP is to compute $t_{k}$ satisfying (1.5) and

1.7 $$ g(x_{k}+t_{k} d_{k})^{T} d_{k}\leq -\sigma \bigl\vert g_{k}^{T} d_{k} \bigr\vert , $$

where $0<\delta <\sigma <1$.

Presently, there are several known formulas for different CG parameters (see [4–10]). One of the most efficient algorithms among the well-known formulas is the PRP [7, 8] defined by

1.8 $$ \beta _{k}^{\mathrm{PRP}}=\frac{g_{k}^{T} y_{k-1}}{ \Vert g_{k-1} \Vert ^{2}}, $$

where $y_{k-1}=g_{k}-g_{k-1}$. From the computational point of view, the PRP algorithm performs better than most CG algorithms, and the convergence result has been established under some line search procedures. However, for a general function, the PRP method fails with regard to the global convergence under the Wolfe line search procedure. This is because the direction of search $d_{k}$ is not descent for a general objective function [4]. This problem inspired numerous researchers to study the global convergence of PRP method under inexact line search. Interestingly, considering the general function, Yuan et al. [11] proved the global convergence of PRP method using a modified Wolfe line search procedure. More practical approaches of the line search have been employed to identify a step-size capable of achieving adequate reduction in the objective function $f(x)$ at minimal cost.

Recently, Rivaie et al. [12] proposed a variant of PRP method by replacing the term $\|g_{k-1}\|^{2}$ in the denominator of PRP with $\|d_{k-1}\|^{2}$ as follows:

1.9 $$ \beta _{k}^{\mathrm{RMIL}}=\frac{g_{k}^{T} y_{k-1}}{ \Vert d_{k-1} \Vert ^{2}}, $$

and showed that the method converges globally under the exact line search. However, Dai [13] pointed out a wrong inequality used in the convergence result of RMIL method and suggested some necessary corrections as follows:

1.10 $$ \beta _{k}^{\mathrm{RMIL}+}= \textstyle\begin{cases} \frac{g_{k}^{T} y_{k-1}}{ \Vert d_{k-1} \Vert ^{2}}, & \text{if } 0\leq g_{k}^{T} g_{k-1}\leq \Vert g_{k} \Vert ^{2}, \\ 0, &\text{otherwise}, \end{cases} $$

and further established the global convergence under the exact line search. Preliminary results have been presented using the same benchmark test problems with different initial guess to illustrate the efficiency of the modified method. More recently, Yousif [14] modified the work of Dai [13] and showed that RMIL+ converges globally under the strong Wolfe line search. For more reference on the convergence analysis of the CG method, please refer to the following references [15–19].

It is worthy to note that the sufficient descent property

1.11 $$ g_{k}^{T} d_{k}\leq \lambda \Vert g_{k} \Vert ^{2}, \quad \lambda >0, $$

plays a crucial role in the convergence analysis of the CG methods including the RMIL method. In this regard, several variants of the CG methods have been defined to satisfy (1.11) independent of the line search technique used.

One of the efficient variants of the CG methods is the three-term CG method where the search direction $d_{k}$ contains three terms. One of the classical three-term methods was proposed by Beale [20], using the coefficient $\beta _{k}^{\mathrm{HS}}$ [5]. The author constructed a new direction of search as follows:

$$ d_{k}=-g_{k}+\beta _{k}d_{k-1}+\gamma _{k} d_{t}, $$

where $d_{t}$ is the restart direction and

$$ \gamma _{k}= \textstyle\begin{cases} 0, & \text{if } k=t+1, \\ \frac{g_{k}^{T} y_{t}}{d_{t}^{T} y_{t}},& \text{if } k>t+1. \end{cases} $$

The performance of this method was improved using an efficient restart strategy developed by McGuire [21]. The first three-term PRP algorithm (TTPRP) was defined by Zhang et al. [22] with the formula given as

$$ d_{k}=-g_{k}+\beta _{k} d_{k-1}+\theta _{k-1} y_{k-1}, $$

where $\beta _{k}$ is the PRP method defined in (1.8) and $\theta _{k}=- \frac{g_{k}^{T} d_{k-1}}{g_{k-1}^{T} g_{k-1}}$. An attractive feature of this method is that the descent condition

1.12 $$ g_{k}^{T} d_{k}\leq - \Vert g_{k} \Vert ^{2}, $$

holds independent of any line search, and the global convergence was established under a modified Armijo line search. Based on the structure of TTPRP, Liu et al. [23] extended the coefficient of RMIL (1.9) to defined a three-term CG method known as TTRMIL with formula as follows:

1.13 $$ d_{0}=-g_{0}, d_{k}=-g_{k}+ \beta _{k} d_{k-1}+\theta _{k} y_{k-1}, \quad k\geq 1, $$

where $\beta _{k}$ is defined by (1.9) and $\theta _{k}=-\frac{g_{k}^{T} d_{k-1}}{\|d_{k-1}\|^{2}}$.

The global convergence of this method was proved under the standard Wolfe line search. However, the proposed TTRMIL method in (1.13) employed the RMIL method; Dai [13] pointed out some errors in the convergence result and suggested some correction given in [14]. Motivated by this, we propose a modification of TTRMIL in the next section. For more references about the three-term CG method, interested readers may refer to [24–27].

The rest of the paper would be structured as follows. In the next section, a modified TTRMIL method is given with its algorithm. The sufficient descent property and the global convergence of the new modification are studied in Sect. 3. Preliminary results based on some unconstrained optimization problems are presented to illustrate the performance of the method in Sect. 4. The proposed modification was extended to formulate a parameterized model for cases of COVID-19 in Sect. 5. In Sect. 6, the application in motion control is presented. Finally, the concluding remark and some recommendations of the study are presented in Sect. 7.

## TTRMIL+ method and its algorithm

Motivated by the comments made by Dai [13] on the convergence of RMIL method, as discussed in the preceding section, we propose a modified TTRMIL, named TTRMIL+, by replacing $\beta _{k}$ in (1.13) with the $\beta _{k}$ given in (1.10) as follows:

2.1 $$ d_{k}= \textstyle\begin{cases} -g_{k}, &k=0, \\ -g_{k}+\beta _{k}d_{k-1}+\theta _{k}y_{k-1}, &k\geq 1, \end{cases} $$

where

2.2 $$ \theta _{k}=-\frac{g_{k}^{T} d_{k-1}}{d_{k-1}^{T} d_{k-1}}. $$

From (1.13) and (2.2), it is obvious that the difference between these two methods is the CG parameter $\beta _{k}$ employed by each method in defining their search directions $d_{k}$. This is a little change that has a great impact in the convergence analysis of RMIL+. It is interesting to note that the TTRMIL+ reduces to the classical RMIL+ method under the exact minimization condition. The following algorithm describes the proposed TTRMIL+.

### Algorithm 1

The modified TTRMIL+ algorithm.

Stage 0. Given $x_{0} \in \mathbb{R}^{n}$, $d_{0}=-g_{0}=-\nabla {f_{0}}$, set $k:=0$.
Stage 1. Check if $\|g_{k}\|\leq \epsilon $, then stop.
Stage 2. Compute $t_{k}$ using (1.5) and (1.6).
Stage 3. Update the new point based on (1.2). If $\|g_{k}\|\leq \epsilon $, terminate the process.
Stage 4. Compute $\beta _{k}$ by (1.10) and update $d_{k}$ by (2.1).
Stage 5. Go to Stage 2 with $k:=k+1$.

The following assumptions are very important and usually required in the convergence analysis of most CG algorithms.

### Assumption 2.1

1. The level set $\Omega =\{x\in \mathbb{R}^{n}|f(x)\leq f(x_{0})\}$ is bounded, where $x_{0}$ is an arbitrary initial point.
2. In some neighborhood *N* of Ω, *f* is smooth and $g(x)$ is Lipschitz continuous on an open convex set *N* that contains Ω such that there exists $L>0$ (constant) satisfying
  2.3 $$ \bigl\Vert g(x)-g(y) \bigr\Vert \leq L \Vert x-y \Vert , \quad \forall x, y \in N. $$

From Assumption 2.1 and [16, 28], it implies that there exist positive constants *γ* and *b* such that

2.4 $$\begin{aligned} \bigl\Vert g(x_{k}) \bigr\Vert \leq \gamma , \quad \forall x_{k}\in \Omega , \end{aligned}$$

2.5 $$\begin{aligned} \Vert x-y \Vert \leq b, \quad \forall x, y \in \Omega . \end{aligned}$$

But the function $f(x)$ decreases as $k \to +\infty $, hence, from Assumption 2.1, the sequence $\{x_{k}\}$ generated by Algorithm 1 is said to be contained in a bounded region. This implies that the sequence $\{x_{k}\}$ is bounded.

The convergence analysis of the new method would be studied in the next section.

## Convergence analysis

In this section, we establish the sufficient descent condition and global convergence properties of the proposed TTRMIL+ method.

The following theorem indicates that the search direction of TTRMIL+ method satisfies the sufficient descent condition.

### Theorem 3.1

*Suppose that the sequence* $\{x_{k}\}$ *is generated by Algorithm *1. *The search direction* $d_{k}$ *defined by* (2.1) *with* $\beta _{k}=\beta _{k}^{\mathrm{RMIL}+}$ (1.10) *satisfies the sufficient descent condition* (1.12).

### Proof

We will prove by induction. For $k=0$ and from (2.1), we have $g_{0}^{T} d_{0}=-\|g_{0}\|^{2}$, so that the sufficient descent condition (1.12) is satisfied. Suppose that (1.12) is true for $k-1$, that is, $g_{k-1}^{T} d_{k-1}=-\|g_{k-1}\|^{2}$. According to the value of $\beta _{k}^{\mathrm{RMIL}+}$ (1.10), we have two cases.

- *Case* 1: $\beta _{k}^{\mathrm{RMIL}+}=0$. Since (1.6), (2.1), (2.2), and $g_{k}^{T}g_{k-1}>\|g_{k}\|^{2}$ hold, we have
  $$ \begin{aligned} g_{k}^{T} d_{k}&=-g_{k}^{T} g_{k}- \frac{g_{k}^{T} d_{k-1}}{d_{k-1}^{T} d_{k-1}} g_{k}^{T} y_{k-1} \\ &\leq - \Vert g_{k} \Vert ^{2}+\sigma \frac{ \Vert g_{k-1} \Vert ^{2}}{ \Vert d_{k-1} \Vert ^{2}}g_{k}^{T} y_{k-1} \\ &=- \Vert g_{k} \Vert ^{2}+\sigma \frac{ \Vert g_{k-1} \Vert ^{2}}{ \Vert d_{k-1} \Vert ^{2}} \bigl( \Vert g_{k} \Vert ^{2}-g_{k}^{T}g_{k-1} \bigr) \\ &\leq - \Vert g_{k} \Vert ^{2}. \end{aligned} $$
- *Case* 2: $\beta _{k}^{\mathrm{RMIL}+}= \frac{g_{k}^{T} y_{k-1}}{\|d_{k-1}\|^{2}}$. From (2.1) and (2.2), we get
  $$ g_{k}^{T} d_{k}=- \Vert g_{k} \Vert ^{2}+ \frac{g_{k}^{T} y_{k-1}}{ \Vert d_{k-1} \Vert ^{2}}g_{k}^{T} d_{k-1}- \frac{g_{k}^{T} d_{k-1}}{ \Vert d_{k-1} \Vert ^{2}} g_{k}^{T} y_{k-1}=- \Vert g_{k} \Vert ^{2}. $$

Hence, the search direction $d_{k}$ defined by the TTRMIL+ method always satisfies the sufficient descent condition (1.12). □

### Remark 3.2

Since the proposed method satisfies the sufficient descent condition (1.12), then, for all $k\geq 0$, we have

3.1 $$ \Vert d_{k} \Vert \geq \Vert g_{k} \Vert . $$

Now, we will establish the global convergence of the TTRMIL+ method by first providing the following lemma to show that the standard Wolfe line search gives a lower bound for the step-size $t_{k}$ as follows.

### Lemma 3.3

*Suppose that the sequence* $\{x_{k}\}$ *is generated by Algorithm *1, *where* $d_{k}$ *is a descent direction and Assumption* 2.1*holds*. *If* $t_{k}$ *is calculated by standard Wolfe line search* (1.5) *and* (1.6), *then we have*

3.2 $$ t_{k}\geq \frac{(1-\sigma ) \Vert g_{k} \Vert ^{2}}{L \Vert d_{k} \Vert ^{2}}. $$

### Proof

From the standard Wolfe condition (1.6) and by subtracting $g_{k}^{T} d_{k}$ in the both sides, and using Lipschitz continuity (2.3), we get

$$ \begin{aligned} (\sigma -1)g_{k}^{T} d_{k} &\leq (g_{k+1}-g_{k})^{T} d_{k} \\ &\leq \Vert g_{k+1}-g_{k} \Vert \Vert d_{k} \Vert \\ &\leq L \Vert x_{k+1}-x_{k} \Vert \Vert d_{k} \Vert \\ &=L t_{k} \Vert d_{k} \Vert ^{2}.\end{aligned} $$

Since $d_{k}$ is a descent direction and also $\sigma <1$, that implies (3.2) is true. □

The following lemma is the Zoutendijk condition [29], which plays an important role in the analysis of the global convergence properties for CG method.

### Lemma 3.4

*Let Assumption* 2.1*hold and* $d_{k}$ *be generated by* (1.10), (2.1), *and* (2.2), *where* $t_{k}$ *is calculated by the standard Wolfe line search* (1.5) *and* (1.6). *Then*

3.3 $$ \sum_{k=0}^{\infty } \frac{(g_{k}^{T} d_{k})^{2}}{ \Vert d_{k} \Vert ^{2}}< + \infty . $$

### Proof

From the standard Wolfe condition (1.5) and (3.2), we have

$$\begin{aligned} f(x_{k} )-f(x_{k}+t_{k} d_{k} )\geq -\delta t_{k} g_{k}^{T} d_{k} \geq \delta \frac{(1-\sigma )(g_{k}^{T} d_{k})^{2}}{L \Vert d_{k} \Vert ^{2}}. \end{aligned}$$

Hence, from Assumption (2.1), we get the Zoutendijk condition (3.3) and hence the proof. □

We present a global convergence results of the proposed TTRMIL+ CG method using the standard Wolfe line search.

### Theorem 3.5

*Suppose that the sequence* $\{x_{k}\}$ *is generated by Algorithm *1, *we have*

3.4 $$ \lim_{k \to \infty } \inf \Vert g_{k} \Vert =0. $$

### Proof

Suppose by contradiction that (3.4) is not true. Then $\forall k\geq 0$, we can find a positive constant *c* so that

3.5 $$ \Vert g_{k} \Vert \geq c. $$

Here, we have two cases.

- *Case* 1: If $\beta _{k}^{\mathrm{RMIL}+}=0$, then based on the Cauchy–Schwarz inequality and from (2.1), (2.2), (2.3), (2.4), (2.5), (3.1), and (3.5), we get
  3.6 $$\begin{aligned} \Vert d_{k} \Vert =& \Vert -g_{k}+\theta _{k} y_{k-1} \Vert \\ =& \biggl\Vert -g_{k}-\frac{g_{k}^{T} d_{k-1}}{d_{k-1}^{T} d_{k-1}} y_{k-1} \biggr\Vert \\ \leq & \Vert g_{k} \Vert + \frac{ \Vert g_{k} \Vert \Vert d_{k-1} \Vert \Vert y_{k-1} \Vert }{ \Vert d_{k-1} \Vert ^{2}} \\ \leq &\gamma +\frac{ \Vert g_{k} \Vert L \Vert x_{k}-x_{k-1} \Vert }{ \Vert d_{k-1} \Vert } \\ \leq &\gamma +\frac{ \Vert g_{k} \Vert L b}{ \Vert d_{k-1} \Vert }\\ \leq& \gamma + \frac{ \Vert g_{k} \Vert L b}{ \Vert g_{k-1} \Vert } \\ \leq &\gamma +\frac{\gamma L b}{c} \triangleq \nu . \end{aligned}$$
  Furthermore, by using (1.12), (3.5), and (3.6), we obtain
  $$ \sum_{k=0}^{\infty }\frac{(g_{k}^{T} d_{k})^{2}}{ \Vert d_{k} \Vert ^{2}}\geq \sum _{k=0}^{\infty }\frac{ \Vert g_{k} \Vert ^{4}}{ \Vert d_{k} \Vert ^{2}}\geq \sum _{k=0}^{ \infty }\frac{c^{4}}{\nu ^{2}}=+\infty . $$
  This is a contradiction with (3.3). Hence, (3.4) holds.
- *Case* 2: If $\beta _{k}^{\mathrm{RMIL}+}=\beta _{k}^{\mathrm{RMIL}}$, then based on the Cauchy–Schwarz inequality and from (1.9), (2.1), (2.2), (2.3), (2.4), (2.5), and (3.1), we obtain
  $$\begin{aligned} \Vert d_{k} \Vert =& \bigl\Vert -g_{k}+\beta _{k}^{\mathrm{RMIL}}d_{k-1}+\theta _{k} y_{k-1} \bigr\Vert \\ \leq & \Vert g_{k} \Vert +\frac{ \vert g_{k}^{T} y_{k-1} \vert }{ \Vert d_{k-1} \Vert ^{2}} \Vert d_{k-1} \Vert + \biggl\Vert -\frac{g_{k}^{T} d_{k-1}}{d_{k-1}^{T} d_{k-1}} y_{k-1} \biggr\Vert \\ \leq & \Vert g_{k} \Vert + \frac{ \Vert g_{k} \Vert \Vert g_{k}-g_{k-1} \Vert \Vert d_{k-1} \Vert }{ \Vert d_{k-1} \Vert ^{2}}+ \frac{ \Vert g_{k} \Vert \Vert d_{k-1} \Vert \Vert g_{k}-g_{k-1} \Vert }{ \Vert d_{k-1} \Vert ^{2}} \\ \leq & \Vert g_{k} \Vert +2\frac{ \Vert g_{k} \Vert \Vert g_{k}-g_{k-1} \Vert }{ \Vert g_{k-1} \Vert } \\ \leq &\gamma +\frac{2\gamma L b}{c}\triangleq \zeta . \end{aligned}$$
  By using the same argument as in Case 1, we obtain (3.4) and the proof is complete. □

## Numerical experiments

In this part, we report the numerical experiments to demonstrate the efficiency of the TTRMIL+ method in comparison with the RMIL [12], RMIL+ [13], PRP [7, 8], and TTRMIL [23] methods. For comparing the computational performance, we consider some test problems from Andrei [30], and Jamil and Yang [31]. Most of initial points are also considered by Andrei [30] and implemented using dimensions starting from 2 to 50,000. The test problems and their initial points are presented in Table 1. The codes were written in Matlab R2019a and run using a personal laptop with specification Intel Core i7 processor, 16 GB RAM, 64 bit Windows 10 Pro operating system. All algorithms are terminated when $\|g_{k}\|\leq 10^{-6}$, and for objective comparison, all the methods are executed under the standard Wolfe line search (1.5) and (1.6) with parameter $\delta =10^{-4}$, $\sigma =0.8$ for the TTRMIL method, and $\delta =0.01$, $\sigma =0.1$ for the RMIL, RMIL+, PRP, and TTRMIL+ methods. The metrics used for comparison include the number of iterations (NOI), the number of function evaluations (NOF), and the central of processing unit (CPU) time.

**Table 1:** List of test problems, dimensions, and initial points

Number	Problems	Dimensions	Initial points
1	Extended White & Holst	1000	(−1.2,1,…,−1.2,1)
2	Extended White & Holst	1000	(10,…,10)
3	Extended White & Holst	10,000	(−1.2,1,…,−1.2,1)
4	Extended White & Holst	10,000	(5,…,5)
5	Extended Rosenbrock	1000	(−1.2,1,…,−1.2,1)
6	Extended Rosenbrock	1000	(10,…,10)
7	Extended Rosenbrock	10,000	(−1.2,1,…,−1.2,1)
8	Extended Rosenbrock	10,000	(5,…,5)
9	Extended Freudenstein & Roth	10,000	(−5,…,−5)
10	Extended Freudenstein & Roth	50,000	(−5,…,−5)
11	Extended Beale	1000	(1,0.8,…,1,0.8)
12	Extended Beale	1000	(0.5,…,0.5)
13	Extended Beale	10,000	(−1,…,−1)
14	Extended Beale	10,000	(0.5,…,0.5)
15	Raydan 1	10	(1,…,1)
16	Raydan 1	10	(−10,…,−10)
17	Raydan 1	100	(−1,…,−1)
18	Raydan 1	100	(−10,…,−10)
19	Extended tridiagonal 1	500	(2,…,2)
20	Extended tridiagonal 1	500	(10,…,10)
21	Extended tridiagonal 1	1000	(1,…,1)
22	Extended tridiagonal 1	1000	(−10,…,−10)
23	Diagonal 4	500	(1,…,1)
24	Diagonal 4	500	(−20,…,−20)
25	Diagonal 4	1000	(1,…,1)
26	Diagonal 4	1000	(−30,…,−30)
27	Extended Himmelblau	1000	(1,…,1)
28	Extended Himmelblau	1000	(20,…,20)
29	Extended Himmelblau	10,000	(−1,…,−1)
30	Extended Himmelblau	10,000	(50,…,50)
31	FLETCHCR	10	(0,…,0)
32	FLETCHCR	10	(10,…,10)
33	Extended Powel	100	(3,−1,0,1,…)
34	Extended Powel	100	(5,…,5)
35	NONSCOMP	2	(3,3)
36	NONSCOMP	2	(10,10)
37	Extended DENSCHNB	10	(1,…,1)
38	Extended DENSCHNB	10	(10,…,10)
39	Extended DENSCHNB	100	(10,…,10)
40	Extended DENSCHNB	100	(−50,…,−50)
41	Extended penalty	10	(1,2,…,10)
42	Extended penalty	10	(−10,…,−10)
43	Extended penalty	100	(1,…,1)
44	Extended penalty	100	(−2,…,−2)
45	Hager	10	(1,…,1)
46	Hager	10	(−10,…,−10)
47	Extended Maratos	10	(1.1,0.1,…,1.1,)
48	Extended Maratos	10	(−1,…,−1)
49	Six hump camel	2	(−1,2)
50	Six hump camel	2	(−5,10)
51	Three hump camel	2	(−1,2)
52	Three hump camel	2	(2,−1)
53	Booth	2	(5,5)
54	Booth	2	(10,10)
55	Trecanni	2	(−1,0.5)
56	Trecanni	2	(−5,10)
57	Zettl	2	(−1,2)
58	Zettl	2	(10,10)
59	Shallow	1000	(0,…,0)
60	Shallow	1000	(10,…,10)
61	Shallow	10,000	(−1,…,−1)
62	Shallow	10,000	(−10,…,−10)
63	Generalized quartic	1000	(5,…,5)
64	Generalized quartic	1000	(20,…,20)
65	Quadratic QF2	50	(0.5,…,0.5)
66	Quadratic QF2	50	(30,…,30)
67	Leon	2	(2,2)
68	Leon	2	(8,8)
69	Generalized tridiagonal 1	10	(2,…,2)
70	Generalized tridiagonal 1	10	(10,…,10)
71	Generalized tridiagonal 2	4	(1,1,1,1)
72	Generalized tridiagonal 2	4	(10,10,10,10)
73	POWER	10	(1,…,1)
74	POWER	10	(10,…,10)
75	Quadratic QF1	50	(1,…,1)
76	Quadratic QF1	50	(10,…,10)
77	Quadratic QF1	500	(1,…,1)
78	Quadratic QF1	500	(−5,…,−5)
79	Extended quadratic penalty QP2	100	(1,…,1)
80	Extended quadratic penalty QP2	100	(10,…,10)
81	Extended quadratic penalty QP2	500	(10,…,10)
82	Extended quadratic penalty QP2	500	(20,…,20)
83	Extended quadratic penalty QP1	4	(1,1,1,1)
84	Extended quadratic penalty QP1	4	(10,10,10,10)
85	Quartic	4	(10,10,10,10)
86	Quartic	4	(15,15,15,15)
87	Matyas	2	(1,1)
88	Matyas	2	(20,20)
89	Colville	4	(2,2,2,2)
90	Colville	4	(10,10,10,10)
91	Dixon and Price	3	(1,1,1)
92	Dixon and Price	3	(10,10,10)
93	Sphere	5000	(1,…,1)
94	Sphere	5000	(10,…,10)
95	Sum squares	50	(0,1,…,0,1)
96	Sum squares	50	(10,…,10)
97	ENGVAL1	50	(2,…,2)
98	ENGVAL1	100	(2,…,2)
99	ENGVAL8	50	(0,…,0)
100	ENGVAL8	100	(0,…,0)
101	QUARTICM	5000	(2,…,2)
102	QUARTICM	10,000	(2,…,2)
103	QUARTICM	15,000	(2,…,2)
104	QUARTICM	20,000	(2,…,2)

**Figure 1:**
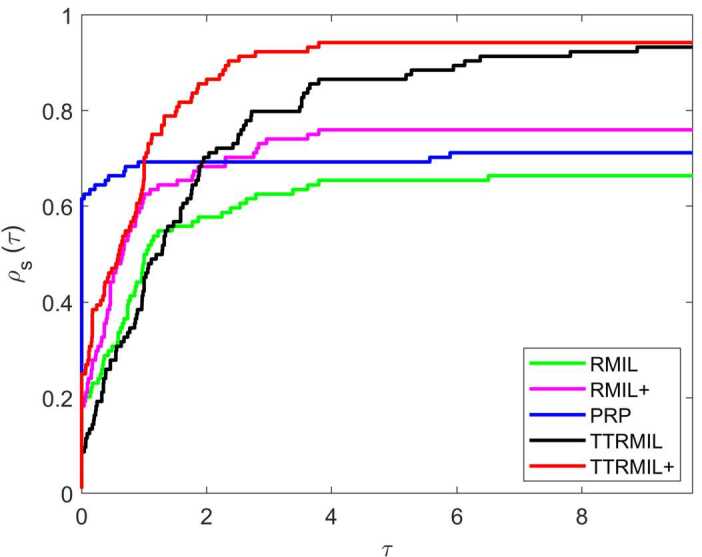
Performance profiles based on NOI

**Figure 2:**
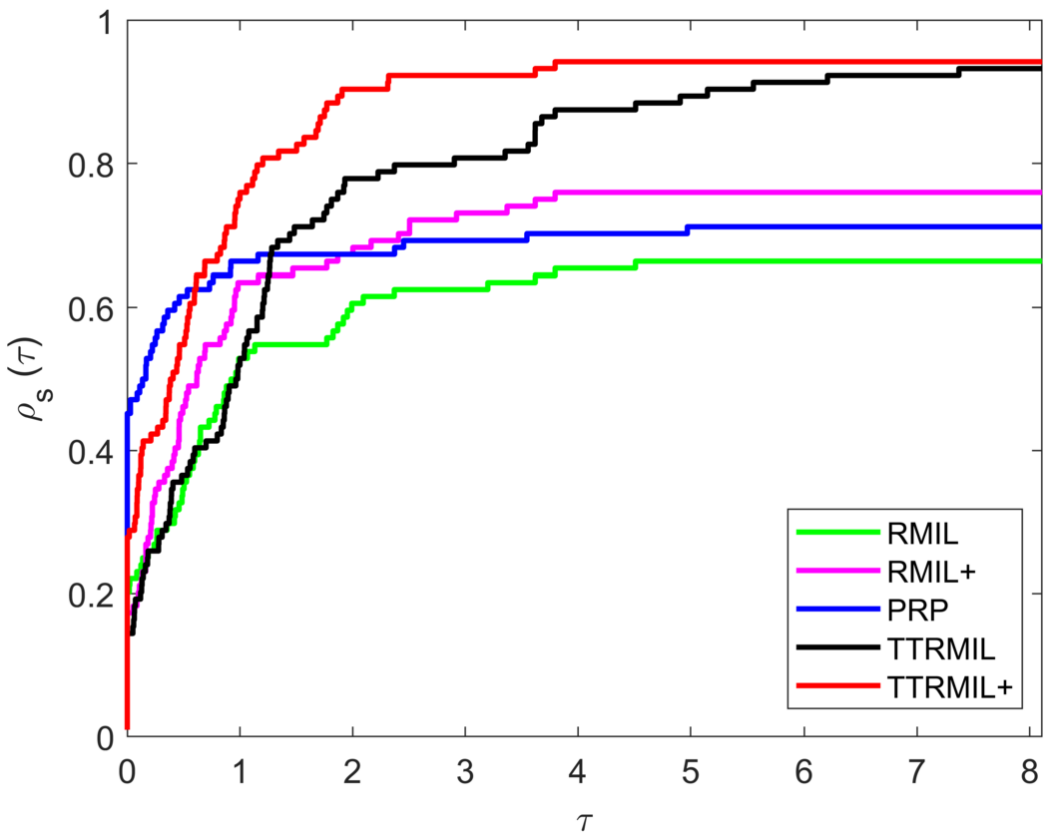
Performance profiles based on NOF

**Figure 3:**
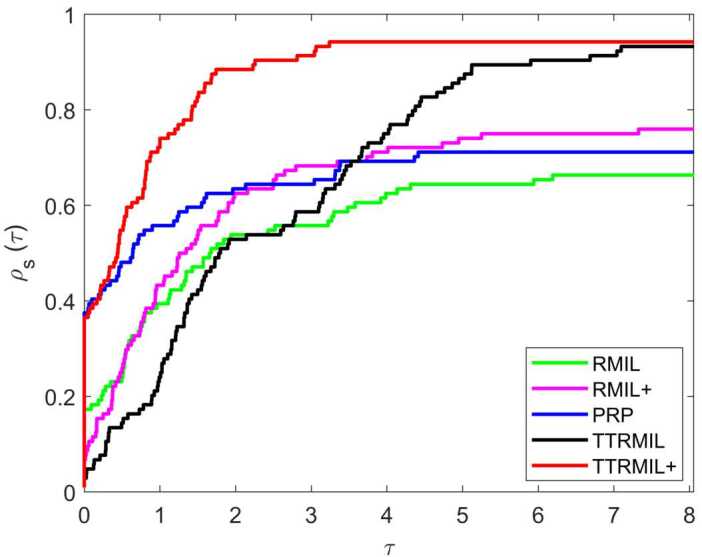
Performance profiles based on CPU time

All numerical results of the RMIL, RMIL+, and PRP methods are listed in Table 2 and those of the TTRMIL and TTRMIL+ methods in Table 3. A method is said to have failed if the NOI is more than 10,000 and the terminating criteria stated above have not been satisfied. The failure is symbolized with ‘F’. We also use the performance profile tool of Dolan and Moré [32] to show the performance profile curve of RMIL, RMIL+, PRP, TTRMIL, and TTRMIL+. The performance profile figures on NOI, NOF, and CPU are presented in Figs. 1, 2, and 3, respectively.

**Table 2:** Numerical results of the RMIL, RMIL+, and PRP methods using weak Wolfe line search

Number	RMIL			RMIL+			PRP		
	NOI	NOF	CPU	NOI	NOF	CPU	NOI	NOF	CPU
1	25	160	0.075	16	102	0.0588	15	104	0.0525
2	F	F	F	F	F	F	21	181	0.0898
3	25	160	0.578	16	102	0.3907	15	104	0.3841
4	F	F	F	38	260	0.9512	22	203	0.7392
5	F	F	F	27	176	0.0488	19	123	0.0377
6	44	227	0.0618	40	243	0.0667	F	F	F
7	F	F	F	32	192	0.3874	19	123	0.231
8	24	126	0.2573	40	195	0.3768	20	136	0.4796
9	F	F	F	11	63	0.1356	8	54	0.1141
10	F	F	F	11	63	0.4922	8	54	0.3937
11	41	137	0.5728	52	191	0.0992	15	69	0.0479
12	56	175	0.0987	F	F	F	9	44	0.0367
13	22	83	0.3537	11	48	0.2153	F	F	F
14	58	182	0.7956	F	F	F	10	47	0.222
15	24	83	0.0015	27	105	0.0026	22	87	0.0021
16	36	143	0.0022	37	170	0.0062	37	157	0.0036
17	110	333	0.0379	109	505	0.0394	74	409	0.032
18	140	435	0.0439	180	841	0.0609	F	F	F
19	12	56	0.0203	6	37	0.0144	F	F	F
20	F	F	F	5	26	0.0145	5	26	0.0139
21	12	56	0.0412	7	40	0.0276	F	F	F
22	8	41	0.0379	9	55	0.0425	13	68	0.0458
23	F	F	F	F	F	F	F	F	F
24	F	F	F	F	F	F	F	F	F
25	F	F	F	F	F	F	F	F	F
26	F	F	F	F	F	F	F	F	F
27	12	43	0.0205	11	44	0.0215	8	34	0.0327
28	10	48	0.0196	7	34	0.0165	6	31	0.0127
29	9	39	0.0942	9	42	0.0952	8	45	0.1075
30	F	F	F	11	50	0.137	8	44	0.1027
31	72	289	0.0036	72	311	0.0084	56	263	0.0055
32	138	712	0.0198	111	548	0.0183	71	376	0.0078
33	F	F	F	70	863	0.0716	3337	10,084	0.7111
34	F	F	F	39	225	0.0443	2312	7053	0.4623
35	8	34	4.81E − 04	54	193	0.0183	15	76	0.0048
36	F	F	F	17	94	0.2085	F	F	F
37	7	22	4.32E − 04	6	22	0.000845	5	19	0.0042
38	8	33	5.90E − 04	8	37	0.0022	8	37	0.0023
39	8	33	0.0038	8	37	0.0093	8	37	0.0044
40	11	52	0.0172	9	43	0.0181	7	37	0.0032
41	F	F	F	27	112	0.0038	31	117	0.0017
42	F	F	F	26	103	0.0021	9	46	6.12E−04
43	26	123	0.0081	19	87	0.0056	12	82	0.006
44	F	F	F	19	89	0.0124	13	87	0.0077
45	F	F	F	F	F	F	F	F	F
46	F	F	F	F	F	F	F	F	F
47	F	F	F	207	923	0.0331	F	F	F
48	40	191	0.0126	31	195	0.0134	25	188	0.004
49	9	39	0.0005699	8	36	0.0053	6	30	0.007
50	10	59	0.0081	11	66	0.0026	F	F	F
51	15	363	0.0034	F	F	F	F	F	F
52	11	226	0.0075	15	400	0.0108	F	F	F
53	2	6	0.0001505	2	6	2.58E − 04	2	6	2.34E−04
54	2	6	0.0105	2	6	2.84E − 04	2	6	4.06E−04
55	1	3	0.0002193	1	3	0.0013	1	3	1.95E−04
56	F	F	F	5	23	0.007	5	23	6.89E−04
57	18	66	0.0024	16	69	0.0028	10	45	0.0011
58	12	46	0.0075	F	F	F	12	59	0.0012
59	14	46	0.0209	11	39	0.0154	F	F	F
60	16	58	0.035	14	59	0.0303	13	51	0.018
61	51	155	0.3235	F	F	F	F	F	F
62	F	F	F	F	F	F	F	F	F
63	24	301	0.0146	F	F	F	F	F	F
64	F	F	F	F	F	F	F	F	F
65	78	265	0.0146	78	280	0.0225	70	250	0.0241
66	78	299	0.0226	77	334	0.0327	58	275	0.0322
67	35	170	0.0046	31	179	0.0023	17	136	0.0012
68	F	F	F	35	265	0.0033	28	243	0.0032
69	21	66	0.0019	22	74	0.0058	23	77	0.0057
70	27	104	0.0155	28	120	0.003	27	117	0.0037
71	F	F	F	7	21	0.0027	F	F	F
72	F	F	F	F	F	F	11	59	0.0019
73	123	369	0.0074	123	369	0.0102	10	30	7.66E−04
74	139	417	0.0139	139	417	0.0123	10	30	8.78E−04
75	69	207	0.0108	69	207	0.0115	38	114	0.0049
76	78	234	0.0093	78	234	0.0104	40	120	0.0073
77	447	1341	0.1754	447	1341	0.1716	131	393	0.0719
78	500	1500	0.2143	500	1500	0.2046	137	411	0.072
79	37	314	0.0274	34	313	0.0254	22	235	0.0161
80	F	F	F	30	296	0.0252	27	296	0.0233
81	60	591	0.099	57	620	0.1127	39	493	0.087
82	3899	12030	1.6296	69	743	0.1158	43	528	0.082
83	14	48	0.0009078	14	53	0.0012	6	28	6.26E−04
84	20	81	0.0144	15	68	0.0013	9	49	9.52E−04
85	773	2468	0.0345	802	2788	0.0517	163	696	0.0138
86	781	2558	0.0395	806	2811	0.0454	113	495	0.0133
87	F	F	F	F	F	F	F	F	F
88	F	F	F	F	F	F	F	F	F
89	773	3091	0.0378	1032	4339	0.0726	148	818	0.2155
90	897	3418	0.0425	669	2819	0.0324	86	372	0.0167
91	42	149	0.0077	F	F	F	F	F	F
92	35	141	0.0196	46	194	0.0083	56	266	0.0063
93	1	3	0.0057	1	3	0.0083	1	3	0.0167
94	1	3	0.0179	1	3	0.0056	1	3	0.0071
95	46	138	0.0123	46	138	0.0152	25	75	0.0057
96	81	243	0.5261	81	243	0.2223	41	123	0.0097
97	23	112	0.0162	47	817	0.0301	22	409	0.0147
98	F	F	F	F	F	F	22	416	0.0251
99	14	46	0.0112	14	63	0.2976	14	78	0.2305
100	14	60	0.0089	F	F	F	F	F	F
101	F	F	F	F	F	F	F	F	F
102	F	F	F	F	F	F	F	F	F
103	F	F	F	F	F	F	F	F	F
104	F	F	F	F	F	F	F	F	F

**Table 3:** Numerical results of the TTRMIL and TTRMIL+ methods using weak Wolfe line search

Number	TTRMIL			TTRMIL+		
	NOI	NOF	CPU	NOI	NOF	CPU
1	93	358	0.1801	23	150	0.0711
2	9929	29,974	12.2849	84	513	0.2698
3	88	342	1.3363	30	181	0.6683
4	4957	14,979	76.0681	45	307	1.4779
5	78	295	0.0782	35	175	0.0473
6	120	467	0.1551	54	313	0.0894
7	108	384	0.7369	30	163	0.2912
8	50	176	0.4424	59	290	0.6383
9	24	120	0.3335	16	87	0.2462
10	24	120	1.0537	27	120	1.0524
11	38	112	0.0976	20	75	0.0503
12	34	101	0.0697	46	148	0.1227
13	59	183	0.8012	24	100	0.6058
14	37	109	0.4909	48	154	1.0408
15	70	164	0.0186	19	65	0.0015
16	122	302	0.0133	39	197	0.0039
17	109	329	0.0205	110	333	0.0402
18	179	539	0.0533	173	541	0.0511
19	369	1110	0.3641	17	80	0.0435
20	414	1216	0.4525	18	83	0.0447
21	488	1419	0.7491	17	80	0.048
22	293	935	0.4831	23	104	0.0648
23	14	39	0.0254	11	30	0.0114
24	19	53	0.0171	13	36	0.0157
25	14	39	0.0223	7	19	0.012
26	19	53	0.0234	13	35	0.0186
27	9	32	0.0359	9	34	0.0144
28	13	58	0.0297	15	60	0.0254
29	10	41	0.1842	9	39	0.1533
30	11	50	0.202	13	57	0.1416
31	73	288	0.0248	72	290	0.005
32	139	699	0.018	141	729	0.0115
33	F	F	F	F	F	F
34	F	F	F	F	F	F
35	31	95	0.014	12	44	0.0023
36	29	102	0.0049	22	100	0.0013
37	8	24	0.0141	5	16	4.05E−04
38	10	40	0.0018	9	35	6.82E−04
39	11	43	0.0039	9	35	0.0032
40	13	73	0.0053	9	47	0.0037
41	27	95	0.0177	23	85	0.014
42	22	83	2.70E − 03	16	66	0.0043
43	F	F	F	26	104	0.0103
44	20	107	0.3942	10	46	0.0066
45	23	60	0.0095	24	63	0.0014
46	34	89	0.0057	32	86	0.0019
47	42	206	0.0132	47	261	0.0057
48	51	213	0.005	50	275	0.0073
49	7	26	0.0071	6	25	3.28E−04
50	8	41	5.62E − 04	9	44	4.61E−04
51	27	85	0.0073	19	275	0.0078
52	F	F	F	23	393	0.0133
53	2	6	1.98E − 02	2	6	4.81E−04
54	2	6	2.60E − 03	2	6	1.71E−04
55	1	3	6.50E − 03	1	3	1.87E−04
56	13	48	5.90E − 03	10	37	9.83E−04
57	21	59	0.0095	17	61	0.0026
58	25	80	9.51E − 04	13	50	7.63E−04
59	27	71	0.371	13	39	0.012
60	40	124	0.1116	19	78	0.0245
61	34	98	2.2351	26	74	0.1941
62	60	195	0.4584	24	82	0.1785
63	F	F	F	F	F	F
64	F	F	F	F	F	F
65	80	261	0.2371	79	268	0.0158
66	90	361	0.019	81	318	0.0186
67	214	703	0.0263	17	94	0.0012
68	125	586	0.0222	54	371	0.0042
69	22	69	0.0138	22	69	0.0013
70	32	132	0.005	27	104	0.0027
71	16	42	1.33E − 02	17	46	6.91E−04
72	14	47	7.42E − 04	23	71	0.0013
73	123	369	1.59E − 02	123	369	0.0064
74	139	417	7.80E − 03	139	417	0.0083
75	69	207	0.0162	69	207	0.0084
76	78	234	0.0182	78	234	0.0105
77	447	1341	0.2032	447	1341	0.1934
78	500	1500	0.188	500	1500	0.2052
79	61	419	0.2168	38	320	0.0215
80	163	701	0.0447	41	324	0.0225
81	1516	5038	0.7924	61	594	0.1043
82	84	683	0.1154	82	762	0.1268
83	10	34	1.03E − 02	15	51	0.0011
84	18	71	1.10E − 03	18	71	0.0013
85	580	1890	0.0407	804	2608	0.0368
86	740	2315	0.0271	777	2471	0.0367
87	29	145	9.60E − 03	9	63	6.19E−04
88	37	185	0.0018	11	77	8.07E−04
89	838	3098	0.0527	683	2426	0.0262
90	567	1924	0.017	493	1847	0.023
91	23	75	1.34E − 02	20	68	6.89E−04
92	36	154	0.0023	39	153	0.0021
93	1	3	0.0723	1	3	0.0064
94	1	3	0.008	1	3	0.0058
95	46	138	0.0085	46	138	0.0071
96	81	243	0.0185	81	243	0.0072
97	24	79	0.0159	24	103	0.0048
98	23	76	0.0107	F	F	F
99	14	46	0.2267	F	F	F
100	F	F	F	15	50	0.0131
101	45	365	0.7906	4	31	0.0886
102	46	381	1.5578	4	31	0.1567
103	46	381	2.2696	4	31	0.2059
104	47	397	2.9721	4	31	0.2747

Let *P* be the set of test problems with $n_{p}$ being the number of test problem. *S* is the set of methods and $n_{s}$ is the number of methods. For each method $s \in S$ and problem $p \in P$, let $j_{p,s}$ denote either NOI, NOF, or CPU time required to solve problem *p* by method *s*. Then the performance profile is defined as follows:

$$ \rho _{s}(\tau )=\frac{1}{n_{p}}\mathit{size}\{p\in P:\log _{2} r_{p,s}\leq \tau \}, $$

where $\tau >0$, and $r_{p,s}$ is the performance ratio that can be obtained by

$$ r_{p,s}=\frac{j_{p,s}}{\min \{j_{p,s}\}}. $$

Generally, the method with the high performance profile value $\rho _{s}(\tau )$ is considered the best method for a given *τ* value. In other words, the method where the curve dominates the very top is the most efficient method compared to the others.

According to Table 2, the RMIL method was able to solve 66% of the problems, RMIL+ 75%, and PRP 71%. Meanwhile, based on Table 3, the TTRMIL method solved 93% of the problems and the proposed TTRMIL+ 94%. In this regard, the TTRMIL+ method is considered a better method when compared to the RMIL, RMIL+, and PRP methods, but competes with the TTRMIL method in terms of NOI, CPU time, and NOF. From the performance profile in Figs. 1–3, we can see that the TTRMIL+ method is efficient and promising with regard to solving unconstrained optimization problems compared to the RMIL, RMIL+, PRP, and TTRMIL methods.

## Application of TTRMIL+ to parameterized COVID-19 model

Coronavirus disease often called COVID-19 is an acute vector-borne disease that surfaced in 2019. This disease is caused by the newly discovered coronavirus (SARS-CoV-2) and can be transmitted through droplets produced when an infected person exhales, sneezes, or coughs. Most people infected by the virus will develop mild to moderate symptoms, such as mild fever, cold, difficulty in breathing, and recover without special treatment. Clinically, as of 3:05 pm CEST, 20 October 2020, a total of 40,251,950 confirmed cases of the COVID-19 with 1,116,131 deaths was recorded from 215 territories and countries around the globe since the disease was first reported in Wuhan, China [WHO].

Recently, numerous studies modeled various aspects of the coronavirus outbreak, and application of numerical methods on some COVID-19 models was also studied. In this paper, we consider the global COVID-19 outbreak from January to September, 2020, model the confirmed cases into an unconstrained optimization problem, and finally apply TTRMIL+ to obtain the solution of the parameterized model.

Consider the following function of regression analysis:

5.1 $$ y=h(x_{1}, x_{2}, \ldots , x_{p}+\varepsilon ), $$

where $x_{i}$, $i=1, 2, \ldots , p$, $p>0$ is the predictor, *y* is the response variable, and *ε* is the error. This type of problem often arises in the fields of management, finance, economics, accounting, physics, and many more. The regression analysis is a statistical modeling tool used to estimate the relationships between a dependent variable and one or more independent variables. To derive the linear regression function, we compute *y* such that

5.2 $$ y=a_{0}+a_{1}x_{1}+a_{2}x_{2}+ \cdots +a_{p} x_{p} +\varepsilon , $$

where the parameters of the regression are defined by $a_{0},\ldots ,a_{p}$. The regression analysis estimates the regression parameters $a_{0},a_{1},\ldots ,a_{p}$ such that the value of the error *ε* is minimized. An instance where the linear regression method is the relationship between *y* and *x* is approximated with a straight line. However, such a case infrequently occurs, and thus, the nonlinear regression process is often used. In this study, we consider the nonlinear regression approach.

To formulate the approximate function, we consider the data from the global confirmed cases of COVID-19 from January to September, 2020. The detailed description of the process follows from the statistics presented in Table 4 which are taken from the data obtained by the World Health Organization [WHO] [33]. We have data for nine months (Jan–Sept), the data for the months would be denoted by *x*-variable and the confirmed cases corresponding to these months would be denoted by the *y*-variable. For fitting the data, we only consider the data for eight months (Jan–Aug), and reserve the data for September for error analysis.

**Table 4:** Statistics of confirmed cases of COVID-19, Jan–Sept, 2020

Monthly data (Jan–Sept) (*x*)	Data of confirmed COVID-19 cases (*y*)	Statistics of COVID-19 in %
1	2010	0.16
2	1852	0.14
3	58,863	4.7
4	74,019	6.0
5	115,577	9.3
6	172,158	13.9
7	293,238	23.6
8	269,338	21.7
9	254,423	20.5

From the above data, we obtain the following approximate function for the nonlinear least square method:

5.3 $$ f(x)=-26{,}029.59+14{,}557.39x+3290.077x^{2}. $$

Function (5.3) is used to approximate the values of *y* based on values of *x* from Jan–Aug. Denoting the number of months by $x_{j}$ and the corresponding confirmed cases by $y_{j}$, then, we can transform the least squares problem (5.3) into the following unconstrained minimization model:

5.4 $$ \min_{x \in \mathbb{R}^{n}} f(x)=\sum _{j=1}^{n} \bigl( \bigl(u_{0}+u_{1} x_{j}+u_{2} x_{j}^{2} \bigr)-y_{j} \bigr)^{2}. $$

The nonlinear quadratic function for the least squares problem is derived using the data utilized from Jan–Aug, 2020, which is further used to formulate the corresponding unconstrained optimization model. Obviously, it can be observed that data $x_{j}$ and the value of $y_{j}$ possess some parabolic relations with the regression parameters $u_{0}$, $u_{1}$, and $u_{2}$ and the regression function (5.4).

5.5 $$ \min_{x \in \mathbb{R}^{2}} \sum_{j=1}^{n} E_{j}^{2}=\sum_{j=1}^{n} \bigl( \bigl(u_{0}+u_{1} x_{j}+u_{2} x_{j}^{2} \bigr)-y_{j} \bigr)^{2}. $$

Using the data from Table 4, we can transform (5.5) to obtain our nonlinear quadratic unconstrained minimization model as follows:

5.6 $$ \begin{aligned} &9u_{0}^{2}+90u_{0} u_{1}+570u_{0} u_{2}-2{,}482{,}956u_{0}+285u_{1}^{2}+4050u_{1} u_{2}\\ &\quad {}-17{,}172{,}778u_{1}+15{,}333u_{2}^{2} \\ &\quad {}-126{,}050{,}318u_{2}+275{,}210{,}100{,}844. \end{aligned} $$

The data considered to generate the unconstrained optimization model are data from Jan–August, and the data for Sept is reserved for computing the relative errors of the predicted data. Applying the proposed TTRMIL+ method on model (5.6) under the strong Wolfe line search, we obtain the following results presented in Table 5.

**Table 5:** Test results for optimization of quadratic model for TTRMIL+

Initial points	NOI	CPU time
(3,3,3)	14	0.03259193087998213
(5,5,5)	13	0.04000198696240659
(21,21,21)	15	0.04062229692033143
(100,100,100)	15	0.04526743733986786

One of the major challenges is computing the values of $u_{0}$, $u_{1}$, $u_{2}$ using matrix inverse [34]. To overcome this difficulty, we implement the proposed TTRMIL+ using different initial points. The computation would be terminated if the following conditions hold.

1. The algorithm fails to solve the model.
2. The number of iterations exceeds 1000. This point is denoted as ‘Fail’.

### Trend line method

**Figure 4:**
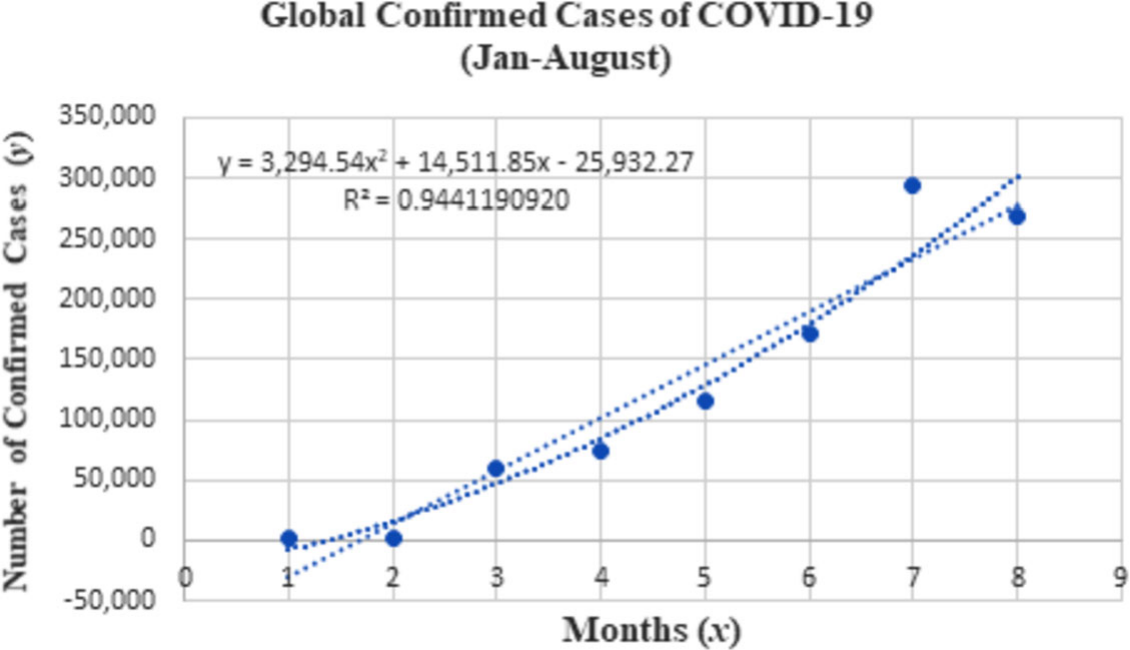
Nonlinear quadratic trend line for confirmed cases of COVID-19

A trend line is a line drawn under pivot lows or over pivot highs to show the prevailing direction of price. In this section, we estimate the data for COVID-19 for a period of nine (9) months using the proposed TTRMIL+ and least squares methods. The trend line is plotted using the Microsoft Excel software based on data from Table 4. The trend line equation appears in a form of nonlinear quadratic equation. Representing the *y*-axis by *y* and *x*-axis by *x*, we obtain the plot presented in Fig. 4 using the actual data from Table 4. Further, to illustrate the efficiency of the proposed method, we compare the approximation functions of TTRMIL+ method with the functions of trend line and least square methods as follows.

The ideal purpose of regression analysis is estimating the parameters $a_{0},a_{1},\ldots ,a_{p}$ such that the error *ε* is minimized. From the results presented in Table 6, it is obvious that the proposed TTRMIL+ CG method has the least relative error compared to the least square and trend line methods which implied that the method is applicable to real-life situations. For other references regarding modeling, analysis, and prediction of COVID-19 cases, one can see [35].

**Table 6:** Estimation point and relative errors for 2020 data

Models	Estimation point	Relative error
TTRMIL+	19,256.790790	0.23786793800
Least square	18,186.200000	0.280239046000
Trend line	18,186.200000	0.280239046000

## Application TTRMIL+ in motion control

This section demonstrates the performance of the proposed TTRMIL+ CG method on motion control of a two-joint planar robotic manipulator. As presented in [36], the following model describes a discrete-time kinematics equation of two-joint planar robot manipulator at the position level

6.1 $$ \Gamma (\mu _{k}) = \eta _{k}, $$

where $\mu _{k}\in \mathbb{R}^{2}$ and $\eta _{k}\in \mathbb{R}^{2}$ denote the joint angle vector and the end effector vector position, respectively. The vector-valued function $\Gamma (\cdot )$ represents the kinematics function which has the following structure:

6.2 $$\Gamma (\mu_{k})=\left[\begin{array}{c} \tau_{1}\cos(\mu_{1})+\tau_{2}\cos(\mu_{1}+\mu_{2})\\ \tau_{1}\sin(\mu_{1})+\tau_{2}\sin(\mu_{1}+\mu_{2}) \end{array}\right],$$

with $\tau _{1}$ and $\tau _{2}$ denoting the length of the first and second rod, respectively. In the case of motion control, at each instantaneous computational time interval $[t_{k}, t_{k+1})\subseteq [0, t_{f}]$ with $t_{f}$ being the end of task duration, the following nonlinear least squares model is to be minimized:

6.3 $$ \min_{\Gamma _{k}\in \mathbb{R}^{2}}\frac{1}{2} \Vert \Gamma _{k}- \widehat{\Gamma }_{k} \Vert ^{2}, $$

where $\widehat{\Gamma }_{k}$ denotes the end effector controlled track.

Similar to the approach presented in [37–39], the end effector, used in this experiment, is controlled to track a Lissajous curve given as

6.4 $${\hat{\Gamma }}_{k}=\left[\begin{array}{c} \frac{3}{2}+\frac{1}{5}\sin(\frac{\pi t_{k}}{5})\\ \frac{\sqrt{3}}{2}+\frac{1}{5}\sin(\frac{2\pi t_{k}}{5}+\frac{\pi }{3}) \end{array}\right].$$

The parameters used in the implementation of the proposed TTRMIL+ CG method are: $\tau _{1}=1$, $\tau _{2}=1$, and $t_{f}=10$ seconds. The starting point $\mu _{0}=[\mu _{1}, \mu _{2}]=[0, \frac{\pi }{3}]^{T}$ where the task duration $[0, 10]$ is divided into 200 equal parts.

**Figure 5:**
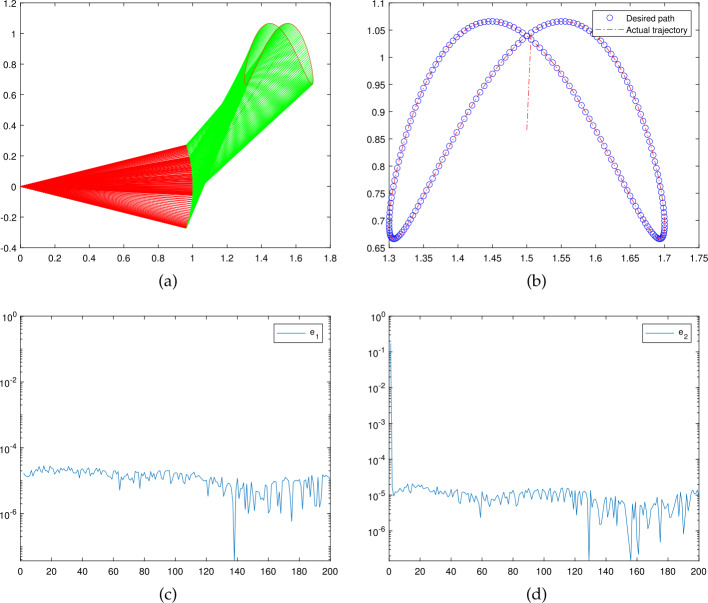
Numerical results generated in the the course of robotic motion control experiment: (**a**) Robot trajectories synthesized by TTRMIL+. (**b**) End effector trajectory and desired path by TTRMIL+. (**c**) Residual error by TTRMIL+ on *x*-axis. (**d**) Residual error by TTRMIL+ on *y*-axis

The results of the motion control experiments are depicted in Figs. 5(a)–5(b). The robot trajectories synthesized by the TTRMIL+ are shown in Fig. 5(a), where the end effector trajectory and the desired path are plotted in Fig. 5(b). Finally, the errors recorded on horizontal and vertical axes by the TTRMIL+ are shown in Figs. 5(c) and 5(d), respectively. Perusing through these figures, it can be seen from Figs. 5(a) and 5(b) that the TTRMIL+ successfully accomplished the task at hand. The error recorded in the course of the task is relatively low as can be seen from Figs. 5(c) and 5(d), which confirms the efficiency of the proposed TTRMIL+.

## Conclusion

This paper presented a modified conjugate gradient method for unconstrained optimization models. The proposed TTRMIL+ method replaced RMIL in TTRMIL with a new modification known as RMIL+. The sufficient descent condition and the convergence proof of TTRMIL+ are studied under the standard Wolfe line search. Some unconstrained benchmark test problems are considered to illustrate the performance of the proposed method. The result obtained showed that the TTRMIL+ method is efficient and promising. The method was further applied to a parameterized COVID-19 model, and the result obtained showed that TTRMIL+ produced a good regression model and thus can be used in regression analysis. Finally, we applied the method to solve a practical problem of motion control. Future work includes studying the new algorithm on nonlinear least squares problems as discussed in [40]. Furthermore, we shall consider other problems in our future research as presented in the following references [41–44].

## Data Availability

Not applicable.

## Abbreviations

CG: conjugate gradient
RMIL: Rivaie, Mustafa, Ismail, and Leong
TTRMIL: Three-term Rivaie, Mustafa, Ismail, and Leong
PRP: Polak–Ribière–Polyak
NOI: Number of iterations
NOF: Number of function evaluations
CPU: CPU time
SARS-CoV-2: Severe acute respiratory syndrome coronavirus 2
COVID-19: Coronavirus disease caused by SARS-CoV-2.
